# How post-translational modifications impact glucocorticoid receptor function in human pathologies

**DOI:** 10.1186/s12964-026-03015-7

**Published:** 2026-06-20

**Authors:** Zélie Bouveret, Ludivine Pruvost, Olivier Trédan, Muriel Le Romancer, Coralie Poulard

**Affiliations:** 1 Université Claude Bernard Lyon 1, Lyon, 69000 France; 2 Inserm U1052, Centre de Recherche en Cancérologie de Lyon, Lyon, 69000 France; 3 UMR5286, Centre de Recherche en Cancérologie de Lyon, CNRS, Lyon, 69000 France; 4 Oncology Department, Centre Leon Bérard, Lyon, France

**Keywords:** Glucocorticoids, Glucocorticoid receptor, Post-translational modifications, Pathology, Cancer

## Abstract

Glucocorticoid receptor (GR) is a member of the nuclear hormone receptor family, which acts as a transcription factor when bound by glucocorticoid (GC) ligands. GR is expressed in nearly all tissue types and regulates essential processes such as inflammation, immune regulation and metabolism. Given its ubiquitous role, GR has frequently been associated with a wide range of illnesses, particularly in the fields of allergy, pulmonary, dermatology, rheumatology, or ophthalmology. It was reported that GCs either contribute to their development or serve as part of their treatment, making them the most prescribed drugs worldwide. GR activity and signaling is finely regulated by a network of post-translational modifications (PTMs). Indeed, PTMs can alter GR behavior and function by modifying its localization, stability, interaction with other proteins and transcriptional activity. Aside from the well-characterized phosphorylation events, additional PTMs are implicated in GR activity and their dysregulation has been described in various diseases. This review provides an integrated overview of current knowledge on GR PTMs, highlighting both mechanistic insights and their relevance in disease. We will present how aberrant PTMs contribute to extremely prevalent diseases, such as cancer, chronic inflammatory diseases, Alzheimer’s disease and other neurological diseases. Special attention will be given to the specific readers of these PTMs and to the enzymes catalyzing these modifications, as they represent promising therapeutic targets.

## Introduction

The term glucocorticoid (GC) reflects both function and origin: *gluco* for the regulation of glucose metabolism and *corticoid* for their production in the adrenal glands. GCs exert their physiological effects by binding to the glucocorticoid receptor (GR), a ligand-dependent transcription factor that is expressed in nearly all tissue types.

Through their properties, GCs are thus involved in a wide range of physiological mechanisms and unsurprisingly also in diseases, either contributing to their development or serving as part of their treatment. The biomedical interest in GCs first emerged in 1950 when Kendall, Reichstein and Hench received the Nobel prize for their groundbreaking work demonstrating their therapeutic efficacy in the treatment of rheumatoid arthritis [1]. In 2017, a retrospective study showed that one in five American adults had received a short-term GC prescription [2]. GCs are currently prescribed in many medical fields, such as in allergy, pulmonary, dermatology, rheumatology and ophthalmology, and were extensively used during the Covid-19 pandemic [2, 3]. In oncology, GCs are used as adjuvant in solid tumors and as primary treatment for some hematologic malignancies.

GR activity is tightly regulated by various post-translational modifications (PTMs) which critically influence its cellular localization, protein stability, interaction with other proteins and transcriptional activity. Aside from the well-characterized phosphorylation events, GR is also subjected to ubiquitylation, acetylation, sumoylation, O-GlcNAcylation, methylation or palmitoylation, impacting many physiological processes. The goal of this review is to provide an overview of the current knowledge on GR PTMs, with a particular emphasis on those that are dysregulated in the context of diseases.

## Glucocorticoid receptor biology

### Glucocorticoid receptor

Glucocorticoid receptor (GR) is a member of the nuclear receptor superfamily and functions as a ligand-activated transcription factor encoded by the *NR3C1* gene, located on chromosome 5 (5q31.3). The *NR3C1* gene consists of nine exons, with exons 2 to 9 encoding the GR protein. Alternative splicing generates several GR isoforms, including GRα and GRβ, which are the predominant variants [4].

GRα is the full-length receptor and transcriptionally-active isoform mediating most GC-dependent responses. Conversely, GRβ lacks agonist-binding capacity due to truncation of the ligand-binding domain, is constitutively nuclear, transcriptionally inactive, and acts as a dominant-negative regulator of GRα [5, 6]. This review focuses on GRα, hereafter referred to as GR. Across various cell types, GR influences approximatively 10% of the human genome, underscoring its broad pleiotropic functions [7]. GR is expressed in nearly all cell types, which reflects its central role in almost all biological functions [8].

**Fig. 1:**
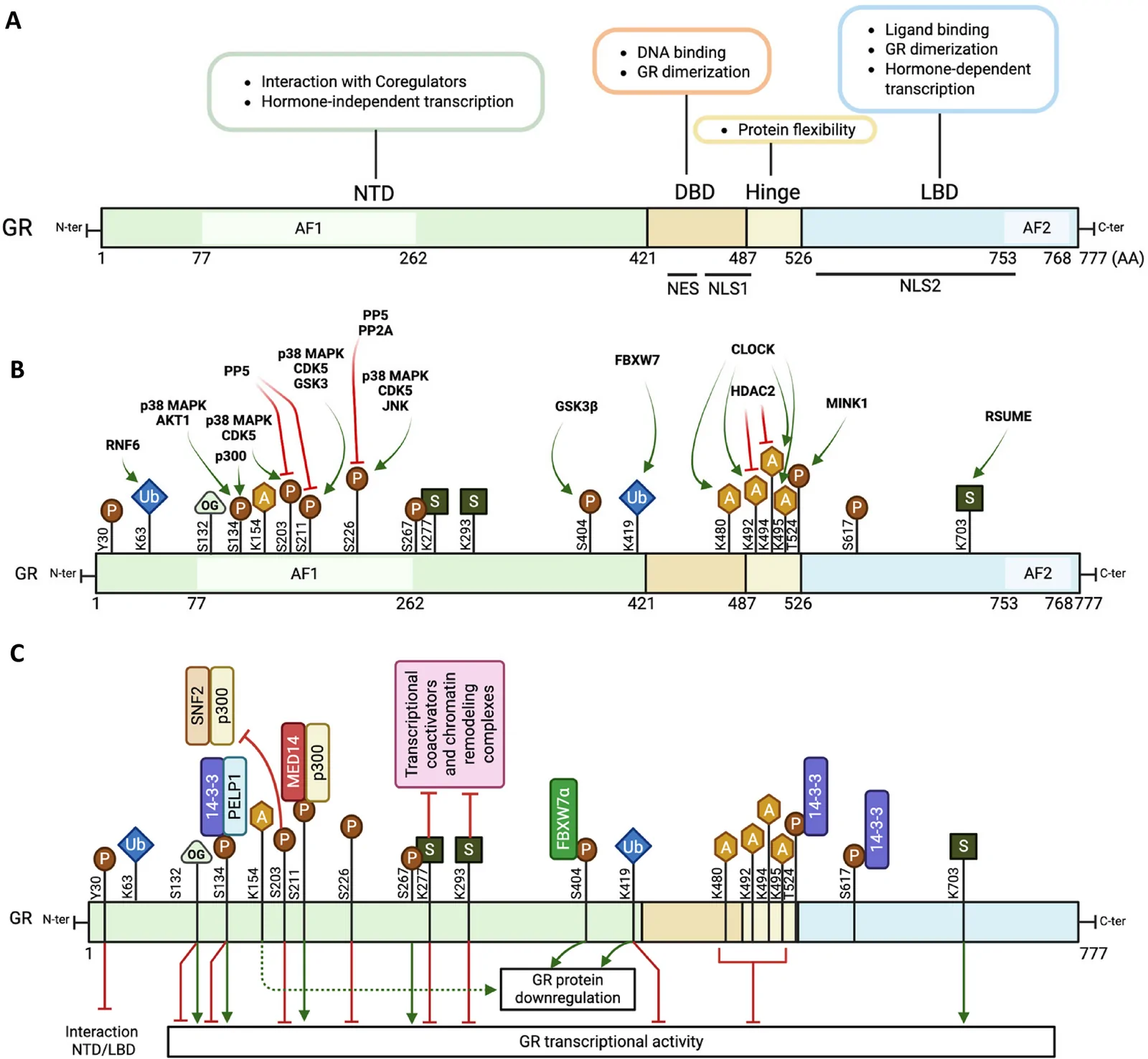
GR PTMs. **A** Domains: NTD: N-terminal domain, DBD: DNA-binding domain, LBD: Ligand-binding domain, AF: Activation function, NES: Nuclear export signal, NLS: Nuclear localization signal. **B** Modified residues are represented on the GR sequence. Green arrows indicate the enzymes responsible for these modifications. Red arrows highlight the phosphatases or histone deacetylases involved. Ub: Ubiquitylation, OG: O-GLycNAcylation, P: Phosphorylation, S: Sumoylation, A: Acetylation. **C** Proteins recruited by specific modifications are annotated on the top of the sequence and the functions associated on the bottom

Structurally, GR exhibits the conserved modular organization typical of nuclear receptors, consisting of four major domains: the N-terminal domain (NTD), DNA-binding domain (DBD), hinge region and ligand-binding domain (LBD) [9, 10] (Fig. 1A). The intrinsically disordered NTD contains activation function 1 (AF1; amino acids 77–262), which enables ligand-independent transcriptional activation and recruits coregulatory proteins and transcriptional machinery. The highly conserved DBD includes two zinc finger motifs responsible for DNA recognition and receptor dimerization, as well as signals governing nuclear trafficking [11, 12]. The hinge region confers conformational flexibility, while the LBD contains activation function 2 (AF2), mediates ligand-dependent GR transcription and receptor dimerization, and harbors the ligand-binding pocket formed by 12 α-helices and 4 β-strands, allowing high affinity binding with GCs [13].

### Glucocorticoid receptor ligands

Glucocorticoids (GCs) were first identified as components of adrenal extracts that decreased the symptoms of patients suffering from adrenal insufficiency, a condition later named Addison’s disease. In the late 1940 s, the isolation by Edward Kendall of cortisol, the predominant GC in humans and its remarkable efficacy in treating rheumatoid arthritis marked a major breakthrough in anti-inflammatory therapy.

#### Natural GCs

Natural GCs are steroid hormones derived from cholesterol and synthesized in the adrenal cortex in response to adrenocorticotropic hormone (ACTH). Cortisol is the predominant GC in humans, whereas corticosterone fulfills this role in rodents. GC biosynthesis involves multiple enzymatic steps, with peripheral metabolism occurring mainly in the liver and kidneys [14]. Local GC availability is tightly regulated by 11β-hydroxysteroid dehydrogenase type 1 (11β-HSD1) enzyme that converts inactive cortisone into active cortisol, while 11β-HSD2 catalyzes cortisol inactivation [15, 16]. A fine tuning of this enzymatic activity is essential, and its dysregulation is associated with various metabolic and inflammatory diseases. Endogenous GC levels are tightly regulated to ensure proper development, function and maintenance of the organs.

The second most abundant GC in the human body is corticosterone, albeit its activity is much lower compared to cortisol and compared to its activity in rodents. Additional GC metabolites, including 5β-dihydrocortisol and tetrahydrocortisol, have been identified [17]. Notably, 5β-dihydrocortisol has been reported to induce apoptosis in breast cancer cells [18].

GC synthesis is regulated by a circadian rhythm, with secretion levels peaking during the active phase of the day [19]. In addition, GC secretion also follows an ultradian rhythm with pulsatile cycles occurring approximately every hour. GC levels also significantly increase in response to physiological or emotional stress, reinforcing their classification as key stress hormones. Growing evidence supports the synthesis of GCs outside the adrenal glands in various tissues, including the intestine, thymus, skin, possibly blood vessels and brain, where GCs primarily exert local effects independent of the central hypothalamic–pituitary–adrenal (HPA) axis [20–23].

In the bloodstream, approximatively 80% of GCs are bound to corticosteroid-binding globulin (CBG) and around 15% to albumin, leaving only 5% in a free, biologically-active form [24]. Proteolytic cleavage of CBG at target tissues facilitates GC release and cellular uptake.

#### Synthetic GCs

Synthetic GCs, such as dexamethasone (dex), prednisolone, prednisone, budesonide, betamethasone or hydrocortisone, are structurally modified derivatives of cortisol designed to enhance GR selectivity, potency and duration of action while limiting side effects [25]. For example, dex is a long-acting GC that is resistant to 11β-HSD2-mediated inactivation, does not bind CBG, and displays high specificity for GR over the mineralocorticoid receptor (MR) [26]. Synthetic GCs are classified according to their duration of action and route of administration, with inhaled formulations commonly used in pulmonary diseases and systemic formulations prescribed for broader inflammatory conditions [27, 28]. GC treatment also exerts feedback regulation on GR expression, with prolonged exposure leading to receptor downregulation [29].

#### Other GR ligands

Aside from classical GCs, alternative GR ligands have been identified. The oxysterol OCDO (6-oxo-cholestan-3,5-diol), also known as cholestane-6-oxo-3,5-diol or Yakkasterone, produced through enzymatic oxidation of cholesterol derivatives, binds the LBD of GR and exhibits oncogenic properties in breast cancer, where it promotes cell proliferation and tumor growth [30].

Additionally, the chemotherapeutic agent cisplatin was shown to directly bind GR at cysteine 622 within the LBD, triggering nuclear translocation and transcriptional activity through a mechanism distinct from the classical GC agonists [31]. These findings expand the spectrum of GR ligands and highlight non-canonical modes of GR activation.

### GR antagonists: use in pathology

While synthetic GCs are used to reduce inflammation or treat specific diseases, other pathologies require antagonizing GR activity. Mifepristone is one of the well described GR antagonists, with a threefold stronger affinity for GR than dex [32]. Mifepristone was approved in 2000 as a potent progesterone receptor (PR) antagonist for the medical termination of pregnancies. In 2012, the FDA expanded its approval to include the treatment of hyperglycemia in patients with Cushing’s syndrome, with a rapid improvement of their condition [33]. However, Mifepristone also disrupts the negative feedback loop of the HPA axis, leading to an increase in ACTH and cortisol [34].

Because the transcriptional activity of GR depends on the specific set of coregulators that are recruited for a specific biological pathway [35], pharmaceutical groups have developed selective GR agonists (SGRA) and modulators (SEGRAM). The ultimate goal is to preserve the antagonist activity of GR, while minimizing its toxicity. Relacorilant (CORT125134, Corcept Therapeutics) is one such antagonist with potent effects in the clinic. Indeed, it is a highly selective GR modulator that competitively antagonizes GC activity without binding progesterone receptor [36]. In mouse models, relacorilant prevented hyperinsulinemia and immunosuppression induced by corticosterone, without significantly affecting the brain [37]. Compared to mifepristone, relacorilant produced only modest disinhibition of the HPA axis and was less potent at modulating ACTH release in pituitary cells [37]. In addition, it has a tissue-specific activity [38]. This may reflect a distinct pattern of GR-coregulator recruitment triggered by relacorilant. Different phase I, II and III clinical trials are ongoing to determine relacorilant efficacy and safety [39–45].

### GR signaling pathways

#### In the absence of ligands

**Fig. 2:**
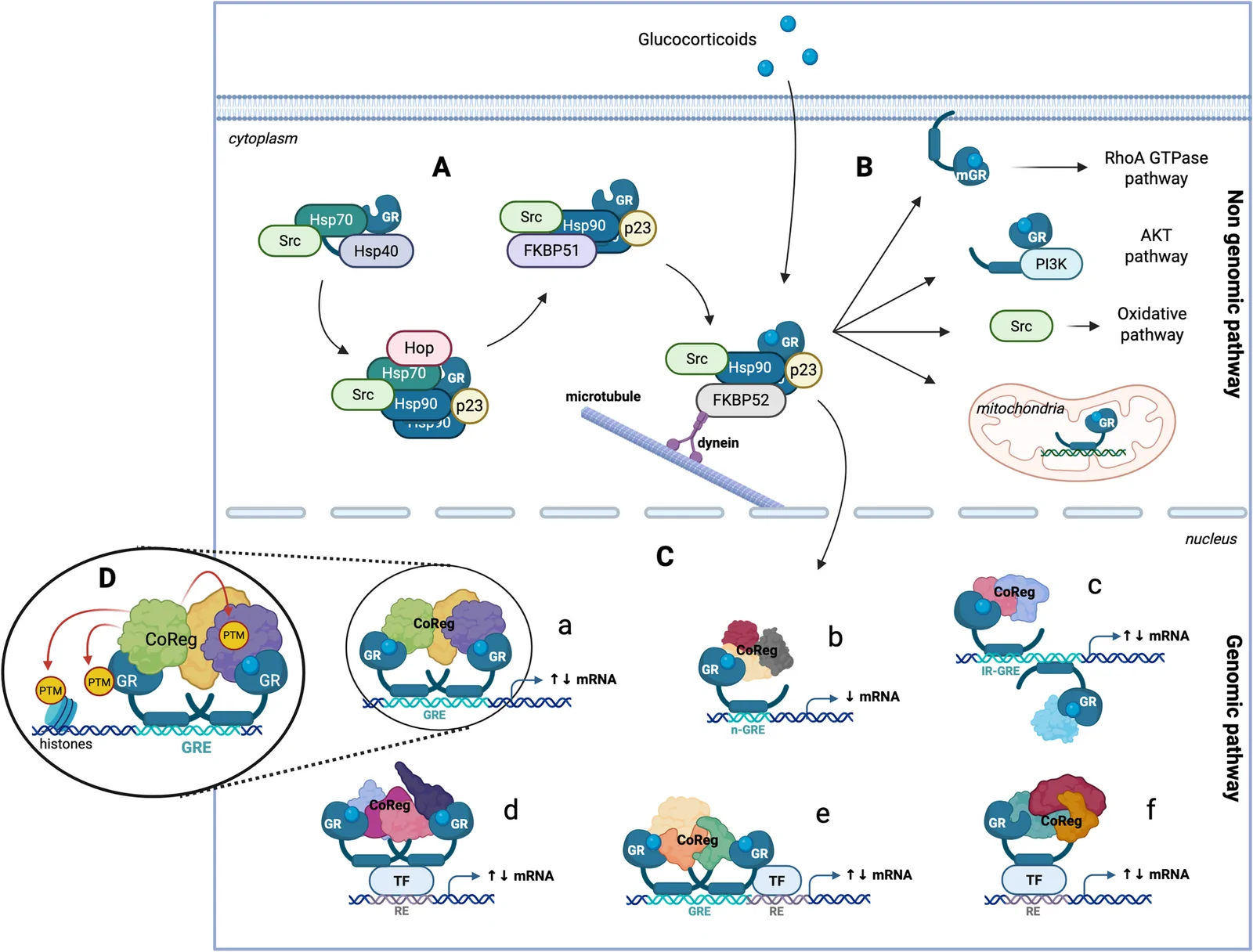
GR signaling. **A** GR maturation and activation before ligand binding. Upon ligand binding, GR follows two major pathways: (**B**) the non genomic pathway, where GR promotes the rapid effects of GCs without nuclear transcription, or (**C**) the genomic pathway that encompasses several modes of GR-dependent transcriptional regulation: (a) binding as a homodimer to canonical GR response elements (GRE), (b) binding as a monomer to GRE half-sites (h-GRE), (c) binding as monomers to inverted-repeat GRE (IR-GRE) in a head-to-tail orientation without dimerizing, (d) tethering to other transcription factors (TFs) on their response elements (RE) and (e) composite binding in which GR directly binds to GREs alongside other TFs. (f) GR also functions in its unliganded form via its binding to TFs. Coregulators are systematically recruited to assist GR in transcriptional regulation. **D** Enlarged panel focusing on enzymatic coregulators responsible for modifying histones, other coregulators (CoReg), or GR itself. PTM: post-translational modifications

In the absence of ligands, GR is predominantly localized in the cell cytoplasm as an inactive monomer within a dynamic multiprotein complex (Fig. 2A). This chaperone complex is essential for GR folding, maturation, ligand binding, activation and subsequent nuclear translocation. The GR-chaperone machinery is composed of heat-shock proteins, including Hsp90, Hsp70 and Hsp40, and other cofactors such as Hop, p23 and immunophilins like FKBP51 and FKBP52 [46–48] (Fig. 2A). Following translation, GR interacts with Hsp70 previously activated by Hsp40. This early folding stage is followed by the transfer of GR from Hsp70 to Hsp90, a transition mediated by the co-chaperone Hop. The recruitment of p23 and FKBP51 to the GR-Hsp90 complex stabilizes a conformation with high affinity for GCs.

Binding of a ligand triggers a conformational change that displaces FKBP51 and FKBP52, exposes the NLS and facilitates its interaction with importins and nucleoporins, which mediate GR translocation to the nucleus via the nuclear pore complex [49–51]. Components of the chaperone complex remain associated with GR during nuclear import and are critical for its efficient translocation [52]. It was demonstrated that FKBP52 recruits the motor protein dynein to the chaperone complex upon ligand binding in order to facilitate GR translocation to the nucleus [53]. The nuclear export of GR is regulated by calreticulin and exportins, which recognize the NES motif on GR. This interaction disrupts GR recruitment to DNA binding sites, terminates transcriptional activity, and facilitates its return to the cytoplasm [54, 55]. GR localization is dynamically regulated, and the balance between its nuclear import and export directly influences the extent and duration of its transcriptional effects. In addition, while the majority of GR functions are nuclear, whereby GR binds to GREs on DNA and modulates gene expression, non-genomic activities have also been described.

#### Non-genomic GR activities

GR mediates rapid non-genomic effects within minutes of exposure to GCs in the cytoplasm, independently of nuclear gene transcription (Fig. 2D). These non-genomic functions have mainly been described in liver and skeletal muscle, though they have been less extensively characterized than the classical genomic effects of GCs. These diverse non-genomic mechanisms contribute to several rapid physiological effects of GCs, such as the rapid negative feedback inhibition of ACTH secretion by the pituitary gland [56], immunosuppression in T cells [57], and vasorelaxation in myocardial and cerebral Ischemia [58]. Research in this field emerged as some of the rapid effects following high-dose intravenous GC administration could not be attributed to genomic GR mechanisms, which take hours or days.

Mechanistically, non-genomic GR signaling involves several distinct pathways. As previously mentioned, in the absence of ligands, GR is localized in the cytoplasm bound to multiprotein complexes that include accessory proteins, such as the Src tyrosine kinase. Upon ligand binding, Src dissociates from the GR complex and triggers the phosphorylation of inducible nitric oxide synthase (iNOS), activating oxidative pathways that result in DNA damage in breast cancer cells [59]. Additionally, GR interacts with phosphoinositide 3-kinase (PI3K) to inhibit the AKT signaling pathway, resulting in the suppression of pro-tumorigenic activities in skin cancer cells [60].

Another significant non-genomic mechanism involves a pool of membrane associated GR (mGR), especially in immune cells such as monocytes and B lymphocytes. In acute lymphoblastic leukemia cells, activation of mGR by GCs triggers early pro-apoptotic signaling events through the modulation of RhoA GTPase pathways [61]. mGR also regulates intercellular communication and cell proliferation of neuronal progenitors via MAPK-dependent phosphorylation of the gap junction protein connexin-43 [62].

Aside from its activity in the cytoplasm and at the cell membrane, GR can also translocate to mitochondria, where it binds GRE-like elements within the mitochondrial genome either alone or with other factors [63, 64]. Mitochondrial GR regulates genes involved in energy metabolism, in order to influence cellular energy and apoptotic response [65]. In addition, the direct interaction of GR with the mitochondrial membrane leads to a disturbance in proton conductance that results in reduced ATP production, crucial for proper cell functions [66].

#### Genomic activities of GR

After approximately 20 min of GR/GC binding, the complex translocates to the nucleus where it homodimerizes and binds to GREs on DNA, leading to the regulation of gene expression (Fig. 2B). Canonical GREs have a 15-base pair imperfect palindromic sequence (5’-AGAACAnnnTGTTCT-3’) composed of two 6-base pair inverted repeats separated by a 3-base pair spacer. GR binds to these GREs as a homodimer in a head-to-tail fashion, with each receptor subunit occupying one half-site. This precise three-nucleotide spacing is essential for GR dimerization on GRE and typically results in transcriptional activation of target genes [11, 13]. Aside from this classical GRE binding, GR also binds DNA as a monomer to GRE half-sites (such as 5’-AGAACA-3’ or its reverse complement) which usually mediates transcriptional repression [67], and on negative GRE (nGRE), defined by the consensus sequence 5’-CTCC(N)_0–2_GGAGA-3’. nGREs are inverted-repeat binding sites (IR-GRE) where two GR monomers bind in a head-to-tail orientation without dimerizing [68, 69]. These nGREs are widespread in the genome and contribute to repression of genes involved in the HPA axis, inflammation and angiogenesis [68].

In the non-classical genomic pathway, GR can also regulate gene expression indirectly via tethering, *i.e.,* by being recruited to DNA through transcription factor (TF) complexes. These tethering sites lack canonical GRE, GRE half-site or nGRE. Notably, TFs from the AP-1, STAT and NF-κB families recruit GR activated by its ligand through tethering, in order to modulate their transcriptional activity [70, 71]. For instance, the interaction between GR, AP-1 and NF-κB is critical for the anti-inflammatory effects of GCs, leading to the transcriptional repression of inflammatory genes [71]. Additionally, GR binds as a homodimer on a GRE and physically interacts with other TFs in a composite manner, thus influencing gene expression [72].

Like many steroid receptors, GR also functions in its unliganded form. Unliganded GR was shown to regulate the expression of over 600 genes. For instance, in mammary epithelial cells, unliganded GR associates with the promoter of the BRCA1 gene, a key tumor suppressor, via the transcription factor GR-binding protein (GABP), leading to an increase in BRCA1 expression [73, 74].

To efficiently regulate transcription, GR recruits a variety of coregulatory proteins (Fig. 2C). including coactivators promoting gene transcription and corepressors inhibiting transcription. However, many coregulators can participate in both activation and repression depending on the context and its partners [35].

Among them, the p160 SRC family member glutamate receptor interacting protein 1 (GRIP1) acts as a platform for the recruitment of other coregulators [75]. GR also interacts with chromatin remodeling complexes like SWI/SNF, which enhance its transcriptional activity [76]. Components of the mediator complex, such as MED1 and MED14, increase GR activity through direct interactions with the GR LBD and AF1 domain, respectively [77]. Corepressors like nuclear receptor corepressor (NCoR) and silencing mediator of retinoic and thyroid receptors (SMRT) serve as docking platforms to facilitate repression [78].

Many coregulators possess enzymatic activities that modify histones, GR or other coregulators (Fig. 2C). For instance, protein acetyltransferases (HATs) such as the CREB-binding protein (CBP) and p300 are recruited by GR following dexamethasone treatment to acetylate histones and promote gene expression [79]. Conversely, histone deacetylases (HDACs), recruited by NCoR or SMRT, repress transcriptional activity [79].

GR also recruits protein methyltransferases like EHMT2, PRMT5, PRMT1 and CARM1, capable of activating or repressing transcription depending on the cell type [80–84]. Additionally, protein demethylases such as KDM1A interact with GR AF1 domain to enhance transcription [85]. These enzymatic coregulators do not only modify histones but can also add PTMs to other coregulators or directly to GR itself, to finely regulate GR response, as detailed in the following section.

## Post-translational GR modifications

Since PTMs are essential for protein function, decoding the PTMs of GR is a necessary step to understand its activity in both normal tissues and disease contexts. In this section, we will focus on the most extensively characterized PTMs of GR (Fig. 1B), while a comprehensive list of all identified modifications is provided in Table 1.

**Table 1:** Modified sites on GR and their functions

Amino Acids	GR domain	Modifications	Inducers	Enzymes	Function	Ref
Y30	NTD	Phosphorylation	ND	ND	Decreases the interaction between NTD and LBD domains	[86]
K63	NTD	Ubiquitylation	ND	RNF6	Increases GR stability	[87]
S132	NTD (AF1)	O-GlcNAcylation	GCs	OGT	Modulates GR transcriptional activity	[88]
S134	NTD (AF1)	Phosphorylation	Stress signals TGFβ1 BDNF	P38-MAPK AKT1	Decreases dex-dependent GR transcriptional activity Binding site of 14–3-3 and PELP1 Increases GR transcriptional activity BDNF-dependent	[89–94]
K154	NTD (AF1)	Acetylation	GCs	CBP/p300	Induces polyubiquitylation and subsequent degradation	[95]
S203	NTD (AF1)	Phosphorylation	GCs, Progest., Cytokines, Taxol …	P38-MAPK JNK ERK1/2 CDK5	Prevents GR from translocating to the nucleus Decreases GR binding with p300 and SNF2	[96–101]
S211	NTD (AF1)	Phosphorylation	GCs Taxol Nocodazol	P38-MAPK CDK5 GSK3 Aurora A	Activates GR transcriptional activity Binding site for MED14 Facilitates recruitment of p300	[96, 98, 102–104]
S226	NTD (AF1)	Phosphorylation	GCs, Progest., TNFα	P38-MAPK CDK5 JNK NLK	Decreases GR transcriptional activity Facilitates nuclear export Facilitates nuclear export Drives GR proteasomal degradation Induces GR sumoylation	[103, 105–108]
S267	NTD	Phosphorylation	BDNF	ND	GR transcriptional activity BDNF-dependent	[94]
K277	NTD	Sumoylation		SUMO-1	Decreases GR ability to recruit transcriptional complexes	[109–111]
K293	NTD	Sumoylation		SUMO-1	Decreases GR ability to recruit transcriptional complexes Transrepression GR-dependent at nGRE	[109–113]
S404	NTD	Phosphorylation	GCs	GSK3β	Regulates GR stability Binding site for FBXW7α Transrepression GR-dependent Induces GR phosphorylation	[114, 115]
K419	NTD	Ubiquitylation	GCs	FBXW7α PELI1	GR degradation	[115–118]
K480	DBD	Acetylation	GCs	CLOCK	Decreases GR binding affinity to GRE	[95, 119]
K492	Hinge	Acetylation	GCs	CLOCK	Decreases GR binding affinity to GRE	[95, 119]
K494	Hinge	Acetylation	GCs	CLOCK	Decreases GR binding affinity to GRE	[95, 119, 120]
K495	Hinge	Acetylation	GCs	CLOCK	Decreases GR binding affinity to GRE	[95, 119, 120]
T524	Hinge	Phosphorylation		MINK1	Binding site for 14–3-3	[121]
S617	LBD	Phosphorylation		ND	Binding site for 14–3-3	[121]
K703	LBD	Sumoylation		SUMO-1 RSUME	Decreases GR ability to recruit transcriptional complexes Modulates GRIP1 function	[109, 122]
ND	ND	Methylation		PRMT5	ND	[123]
ND	ND	Palmitoylation			Non genomic control of GnRH signaling	[124]

GR PTMs were classified by numeric order. Each type of modification is represented by a different color

*ND* Not determined, *Progest* Progesterone, *OGT* O-linked N-acetylglucosamine transferase, *NTD* N-terminal domain, *AF1* Activation function 1, *LBD* Ligand binding domain

### Phosphorylation

Phosphorylation consists in the addition, by kinase enzymes, of phosphate groups to serine, threonine, or tyrosine residues. Since the 1980 s, it is well known that GR is hyperphosphorylated following ligand binding [125, 126]. Most of the phosphorylation sites are localized within the GR AF1 domain, and some of them form a docking site for the recruitment of proteins; these actors are called readers (Fig. 1C).

#### S134

Phosphorylation of GR at serine 134 (pS134-GR) is catalyzed by AKT1 and p38 MAPK. It occurs independently of GCs and is triggered by cellular stress signals such as glucose deprivation, hydrogen peroxide (H_2_O_2_), ultraviolet (UV) irradiation or heat shock [89, 90]. In the absence of GCs, pS134-GR has minimal impact on global gene transcription. In U2OS cells, microarray analysis revealed that only 20 out of 44,000 genes were affected. However, following GC treatment, pS134-GR becomes critical for transcriptional regulation, influencing the expression of 889 genes in U2OS cells and more than 200 dex-dependent genes in HCT116 cells, as shown by RNA-sequencing [89, 91]. The direct effect of stress signals alone on pS134-GR-mediated gene regulation was unfortunately not assessed in these analyses.

Mechanistically, pS134-GR creates a docking site for the phospho-serine/threonine-binding proteins 14–3-3ζ and 14–3-3σ, in order to influence its subcellular localization and its transcriptional activity in a context- and gene-specific manner [89, 91]. In response to dex, pS134-GR recruits 14–3-3σ, which retains GR in the cytoplasm and decreases its transcriptional response [91, 92]. At the level of specific genes, the interaction between pS134-GR and 14–3-3 modulates GR transcriptional output in a stimulus-dependent manner. Upon oxidative stress in addition to GCs, GR binding to the GILZ promoter remains unchanged. In contrast, the same stress signal decreases both GR and 14–3-3ζ binding at the IGFBP1 promoter, reducing IGFBP1 transcription, an effect abolished in a non-phosphorylated S134A GR mutant, underscoring the functional importance of this phosphorylation [89]. In addition, Lange’s group demonstrated that pS134-GR is a docking site for the coactivator PELP1 (Proline-, glutamic acid- and leucine-rich protein 1) upon dex treatment [93]. PELP1 inhibition impairs its interaction with GR and the subsequent induction of the breast tumor kinase gene (Brk/PTK6).

Thus, p134-GR, through a dynamic interplay with 14–3-3 proteins and coactivator PELP1, serves as a central regulatory node linking stress signaling, subcellular localization, and GR function at the chromatin level.

#### S203

Phosphorylation of GR at S203 (pS203-GR) is mediated by several kinases, including CDK5, p38 MAPK, ERK1/2 and JNK, in response to a variety of stimuli such as GCs, mineralocorticoids, progesterone, cytokines such as IL-13, as well as drugs like taxol and nocodazole [96–100, 105]. This broad range of inducers suggests a non-selective steroidal effect on GR phosphorylation.

Following different kinases, cell models, and culture conditions, changes on pS203-GR were different [96, 127, 128]. However, most of the studies have shown that upon GC treatment, pS203-GR remains cytoplasmic in various cell lines, and could be a signal for other phosphorylation events [96, 97, 101, 128, 129]. In addition, in airway smooth muscle, authors identified that pS203-GR mediated by p38-MAPK inhibits GR activity by preventing its nuclear translocation. Indeed, S203A mutation, which blocks phosphorylation, enhances GR-driven transcription [97].

Additionally, pS203-GR could negatively regulate GR by limiting its interaction with coregulators. Indeed, in the HCT116 colon cancer cell line, CDK5-mediated phosphorylation of S203 suppresses GR transcriptional activity by reducing the recruitment of essential cofactors such as p300 and SNF2 [98].

#### S211

Phosphorylation of GR at S211 (pS211-GR) is a well-established marker of GR activation and plays a central role in the regulation of its transcriptional activity. This modification is induced by several kinases, including CDK5, p38 MAPK, Aurora kinase A and GSK3, and reversed by the serine/threonine phosphatase PP5 or protein phosphatase 2 A (PPP2CA) in complex with striatin-3 [98, 102, 130–134]. GCs such as dex, cortisol and to a lesser extent deoxycorticosterone, trigger pS211-GR within one hour of treatment, promoting GR nuclear translocation and chromatin binding at the GREs [135].

Functionally, pS211-GR enhances the transcription of a subset of GC-responsive genes [103]. Mutation of S211 to alanine, reduces GR-mediated transactivation without affecting GR stability, while the phospho-mimetic mutation S211D increases the interaction of GR with coactivators like MED14 [105]. Similarly, pS211-GR facilitates the recruitment of the transcriptional coactivator p300, in order to activate GR transcriptional activity [132]. Various signaling proteins were shown to affect pS211-GR, such as Pin1, GSK3 or PP1α [104, 136].

Interestingly, p38 MAPK inhibition increases pS211-GR, GR translocation and GR transcriptional activity in ASM cells [131]. In neural progenitor stem cells (NPSC), loss of Caveolin-1 reduces pS211-GR and impairs GR recruitment to the promoter of key target genes (*e.g.,* FKBP5, RhoJ, SGK1), highlighting its functional role in the regulation of cell growth [137]. Pro-inflammatory cytokines (*e.g.,* TNFα/IFNγ) suppress pS211-GR and GR activity by upregulating PP5, whereas IL-13 enhances pS211-GR and GR-mediated transcription when combined to dex [96, 131].

Beyond ligand stimulation, in response to microtubule-targeting agents, like taxol or nocodazole, pS211-GR induced by Aurora kinase A accumulates during mitosis, and drives ligand-independent transactivation [99, 102].

In conclusion, pS211-GR is a dynamic regulatory hub, that integrates multiple upstream signals to finely regulate GR transcriptional responses in both ligand-dependent and -independent contexts.

#### S226

Phosphorylation of GR at S226 (pS226-GR) is catalyzed by multiple kinases, including c-Jun N-terminal kinase (JNK), p38 MAPK, CDK5 and Nemo-like kinase (NLK) [98, 106–108]. This modification occurs at a basal level in the absence of ligand. GR ligands such as dex, cortisol, deoxycorticosterone, progesterone and synthetic progestins were shown to stimulate pS226-GR [103, 105]. pS226-GR plays a critical role in modulating GR transcriptional activity through distinct mechanisms depending on ligand availability.

In the presence of GCs, pS226-GR accumulates in the nucleus and is recruited to the GREs [105, 135]. Phosphorylation at S226 has been shown to reduce the efficacy, but not the potency, of GR-mediated transactivation on a GRE promoter, a finding confirmed across a wide panel of GR ligands [103, 105]. Phosphorylation of S226, similarly to S203 and S211, determines the degree of coregulator GRIP1 recruitment, providing a possible mechanistic explanation for how phosphorylation modulates transactivation efficacy [138]. Furthermore, dex-induced phosphorylation at S226 has been implicated in the selective inhibition of transrepression on certain genes, namely on AP-1 and NF-κB promoters, suggesting that such modifications may impair the ability of GR to interact with these transcription factors [103].

Conversely, upon ligand withdrawal, pS226-GR facilitates nuclear export via exportin 1/CRM1, leading to transcriptional downregulation [106]. In addition, it was recently demonstrated that NLK interacts with GR and phosphorylates it on S226, resulting in ubiquitin-dependent proteasome degradation of GR.

PP5 was shown to dephosphorylate pS226-GR, and phosphatase inhibition (*e.g.,* okadaic acid, calyculin A) results in an increase of pS226-GR [139].

#### S404

Phosphorylation of GR at S404 (p404-GR), catalyzed by glycogen synthase kinase 3 beta (GSK3β), plays a dual role as it regulates GR stability and transcriptional activity [114, 115]. Phosphorylation at S404 creates a recognition site for the E3 ligase component FBXW7α, promoting GR ubiquitylation and proteasomal degradation [115].

Beyond protein stability, pS404-GR also influences GR transcriptional function. While phosphorylation at S404 does not affect nuclear import of the GR following dex treatment, it may influence GR export back to the cytoplasm [114]. In addition, lack of S404 phosphorylation enhances GR-mediated gene regulation and alters the spectrum of GR target genes, suggesting that GSK-3β activity is a key modulator of GR transcriptional responses to GCs. This phosphorylation also affects the ability of GR to repress NF-κB. Indeed, blocking S404 phosphorylation increases GR interaction with the p65 subunit after dex treatment. While both GR WT and GR S404A interact similarly with GRIP1, the S404A mutant shows reduced binding to the coactivator CBP/p300, suggesting that S404 serves as a regulatory site for coregulator recruitment and GR-mediated transrepression [114].

### Ubiquitylation

GR is ubiquitinylated on K419 after dex treatment to trigger its proteasomal degradation. Mutation of this residue (K419A) reduces GR degradation. This leads to an increase in GR stability and enhances its transcriptional activity [116]. A recent study showed that GR ubiquitylation occurred predominantly in the nucleus [140]. This process is tightly regulated by phosphorylation at S404, which serves as a recognition motif for FBXW7α, that promotes GR ubiquitylation [115]. Interestingly, GR dimerization facilitates S404 phosphorylation and subsequent FBXW7α interaction, indicating that GR activation and dimerization are prerequisites for GR turnover [117]. Additionally, β-arrestin-1 stabilizes GR by preventing the transcriptional upregulation of the E3 ligase Pellino-1 (PELI1), which catalyzes GR ubiquitylation and targets it for degradation [118]. Loss of β-arrestin-1 enhances GR turnover and shortens the GC response, revealing complementary pathways, via FBXW7α and PELI1, through which GR stability and signaling are finely regulated.

GR was also shown to be polyubiquitinylated on the K63 residue by the RING-type ubiquitin ligase RNF6 [87]. Surprisingly, rather than targeting GR for degradation, this modification stabilizes the receptor and boosts its transcriptional activity. Mechanistically, authors showed that RNF6 is able to bind GR at the LBD, and subsequently activates GR.

### Sumoylation

Though sumoylation is a similar process to ubiquitylation, it does not target proteins for degradation. It regulates processes like protein–protein interactions and subcellular localization.

GR is modified by sumoylation at three conserved sites on residues K277 and K293 in the N-terminal domain, and K703 in the LBD [109]. K277 and K293 serve as the primary SUMO-1 conjugation sites and are crucial for the regulation of GR functions [109]. Experimental evidence shows that the mutation of these sites significantly increases chromatin accessibility at GRE and subsequently the expression of target genes [110, 111]. These effects are recapitulated pharmacologically by the SUMO E1 enzyme inhibitor ML-792, which mimics sumoylation deficiency. ML-792 enhances the recruitment of GR on chromatin and its transcriptional response [111, 141].

Proteomic analyses have shown that loss of sumoylation enhances the association of GR with nearly 100 proteins, including key coactivators such as NCOA1, chromatin remodelers like SMARCA2, BRD2 and CHD1 and transcription factors, such as JUN and ATF3 from the AP-1 complex. This suggests that sumoylation decreases the ability of GR to recruit transcriptional coactivators and chromatin-modifying complexes, thereby restricting enhancer activity [111].

In addition to its role in enhancer regulation, sumoylation at K293 is essential for GR-mediated transrepression at nGREs [112, 113]. This modification is required for the formation of a repressive complex between GR and the corepressors SMRT/NCoR1 and HDAC3, which collectively mediate the repression of pro-inflammatory TFs such as NF-κB and AP-1 [112, 113]. Sumoylation is essential not only for limiting GR activation but also for enabling its anti-inflammatory functions through tethered indirect transrepression mechanisms.

Although sumoylation of the K703 site in the LBD was previously thought to have a minimal impact on GR function [109], recent findings using rat GR vector reveal that RSUME (RWD-containing SUMOylation enhancer), a sumoylation enhancer upregulated under stress conditions, significantly increases GR sumoylation on K721 (K703 on human GR) and transcriptional activity through this site, by modulating GRIP1 coactivator function [122]. Moreover, RSUME is essential for GR sumoylation induced by heat shock.

Interestingly, an interplay between sumoylation and phosphorylation has been described. Phosphorylation of GR at S226 and S404 facilitates sumoylation at K277 and K293, suggesting that phosphorylation serves in that context as a priming signal for sumoylation [142].

### Acetylation

GR undergoes acetylation at multiple residues in order to regulate GR transcriptional activity and stability. The acetyltransferase CLOCK targets four lysine residues within the GR hinge region (K480, K492, K494 and K495), while CBP/p300 acetylates K154 in the N-terminal domain [95, 119]. Acetylation of K154 is induced by GCs and reversed by the NAD^+^-dependent deacetylase SIRT1 [95].

Acetylation of the hinge lysine residues by CLOCK reduces GR binding affinity to GREs and subsequently decreases transcriptional activity. Consistently, mutations that impair CLOCK acetyltransferase function enhance GR recruitment to GREs [119].

The histone deacetylase HDAC2 selectively removes acetyl groups from K494 and K495, a modification essential for GR interaction with the NF-κB complex, in order to repress pro-inflammatory gene expression [120].

While both wild-type GR and K154R mutant display similar patterns of target gene activation during short-term GC treatment, differences appear upon prolonged exposure [95]. K154R mutation leads to impaired polyubiquitylation and reduced proteasomal degradation. It results in an increase in GR protein stability, GR recruitment on chromatin, and transcriptional activation [95]. This data highlights a crosstalk between acetylation and ubiquitylation where acetylation at K154 modulates both the duration and magnitude of the GC response by regulating GR turnover.

### Other PTMs

Recently, Wang et al. demonstrated that GR can be O-GlcNAcylated at S132 by O-linked N-acetylglucosamine transferase (OGT) [88]. Interestingly authors showed that GR phosphorylation at S211 and S226 are events preceding response to dex treatment, in order to promote O-GlcNAcylation and the transcriptional program. A link between OGT and GR was previously suggested as it was demonstrated that OGT plays a key role in GR-mediated transrepression. Indeed, ligand-bound GR recruits OGT into a multi-protein repression complex, enhancing the ability of GR to repress NF-κB-regulated transcription [143].

GR is also methylated by PRMT5, the major arginine methyltransferase responsible for symmetric dimethylation of arginine residues, in the breast cancer cell line MCF-7. Even though the specific methylation site has not yet been identified, this modification may contribute to important physiological processes [123, 144].

Palmitoylation is a PTM in which a 16-carbon fatty acid called palmitate, is covalently attached to an internal cysteine residue via a thioester bond. Because ERα, PR and AR are known to undergo S-palmitoylation [145], to promote membrane localization of steroid receptors and facilitate rapid non-genomic signaling, it has been proposed that GR might also be modified in this way. However, the literature remains contradictory. Studies reported that GR is not palmitoylated [146, 147], while others claimed that both endogenous and overexpressed GR can be palmitoylated. Authors suggest that negative results would be due to low sensitivity of metabolic-labeling assays performed in different cell models [124]. Importantly, this study also showed that GR palmitoylation does not occur at the predicted consensus cysteine residue, suggesting that, unlike other steroid receptors, GR may lack a conserved palmitoylation motif. Additional work is necessary to identify its potential modification site.

## Post-translational GR modifications dysregulated in pathologies

As PTMs regulate GR activity, their description and a better understanding of their involvement in diseases may improve treatment efficacy (Table 2). GR plays central roles in development, cell survival and apoptosis, metabolic adaptation, immune and inflammatory responses and the maintenance of vascular tone, making its regulation by PTMs particularly important. Enzymes catalyzing these PTMs therefore represent promising therapeutic targets, as they offer opportunities to selectively modulate GR signaling (Table 2). Extensive information is available regarding GR PTMs in cancer, where PTM patterns have been linked to tumor progression, therapeutic resistance and altered transcriptional programs. GR phosphorylation in inflammatory and neurological disorders has also extensively been documented, although these data remain largely descriptive rather than mechanistic. In contrast, very limited information is currently available on GR PTMs in metabolic or reproductive diseases, highlighting a major knowledge gap that warrants further investigation.

**Table 2:** GR PTMs involved in pathologies

PTMs	Pathologies	Status	Effects	Potential targets	Models	Ref
ubK63-GR	MM	ND	Promotes cell proliferation and survival	RNF6	HCL	[87]
O-GlcNAc-S132-GR	Endometrial cancer	ND	Promotes immune evasion and immunotherapy resistance	OGT	HCL	[88]
pS134-GR	T-ALL	ND	GR cytoplasmic localization: Resistance to GCs	AKT1	HCL	[90]
	TNBC	↑	Promotes aggressiveness and metastasis	p38 MAPK	HCL, MM, HS	[93, 148–150]
	AD	↓	Tau hyperphosphorylation and cognitive functions	DUSP1	MM	[94, 151]
pS203-GR	B-ALL	↑	Decreases GCs response	ERK2	HCL	[152]
pS211-GR	COPD	↑	GC insensitivity	p38 MAPK	HS	[153]
	SA	Not Ind	GC insensitivity	PP5	HS	[154]
	Epilepsy	↓	Lack of anti-inflammatory response	ANXA1	MM	[155]
	PTSD	↓	Decreases GR nuclear translocation and gene expression	⊥ GR-FKB	MM	[156]
	BD	↑	ND	ND	HS	[157]
	CLL	↓	Decreases GCs response	PLCγ	HCL, HS	[130, 158]
	ERα + BC	↓ by E2	Suppress GR anti-proliferative effect	ND	HCL	[134]
pS226-GR	SA	↑	GC insensitivity	p38 MAPK	HCL, HS	[107, 159–164]
	MDD	↑	GC resistance	ND	HS	[165]
	B-ALL	↑	Decreases GCs response	ERK2	HCL	[152]
	CLL	↑	Decreases GCs response	PLCγ	HS	[158]
pS267-GR	AD	↓	Tau hyperphosphorylation and cognitive functions	DUSP1	MM	[94]
SUMO-K277 SUMO-K293 SUMO-K703	ERα + BC	ND	Inhibits ERα-dependent cell proliferation	ND	HCL	[166]
acK494-GR acK495-GR	COPD	↑	Decreases association of GR with NF-κB complex, and subsequent increase of inflammation	HDAC2	HCL	[120, 167]
SUMOs-GR	B-ALL	ND	Decreases GC response	SUMO inh	HCL	[141, 168]
Ub-GR	T-ALL	↑	Decreases GC response	FBXW7	HCL	[115]

↓ Decreased, ↑ Increased

*Not Ind* Not induced by GCs, *inh* Inhibitor, *HCL* Human cell lines, *HS* Human samples, *MM* Mouse models, *PBMC* Peripheral blood mononuclear cells

### Cancers

**Fig. 3:**
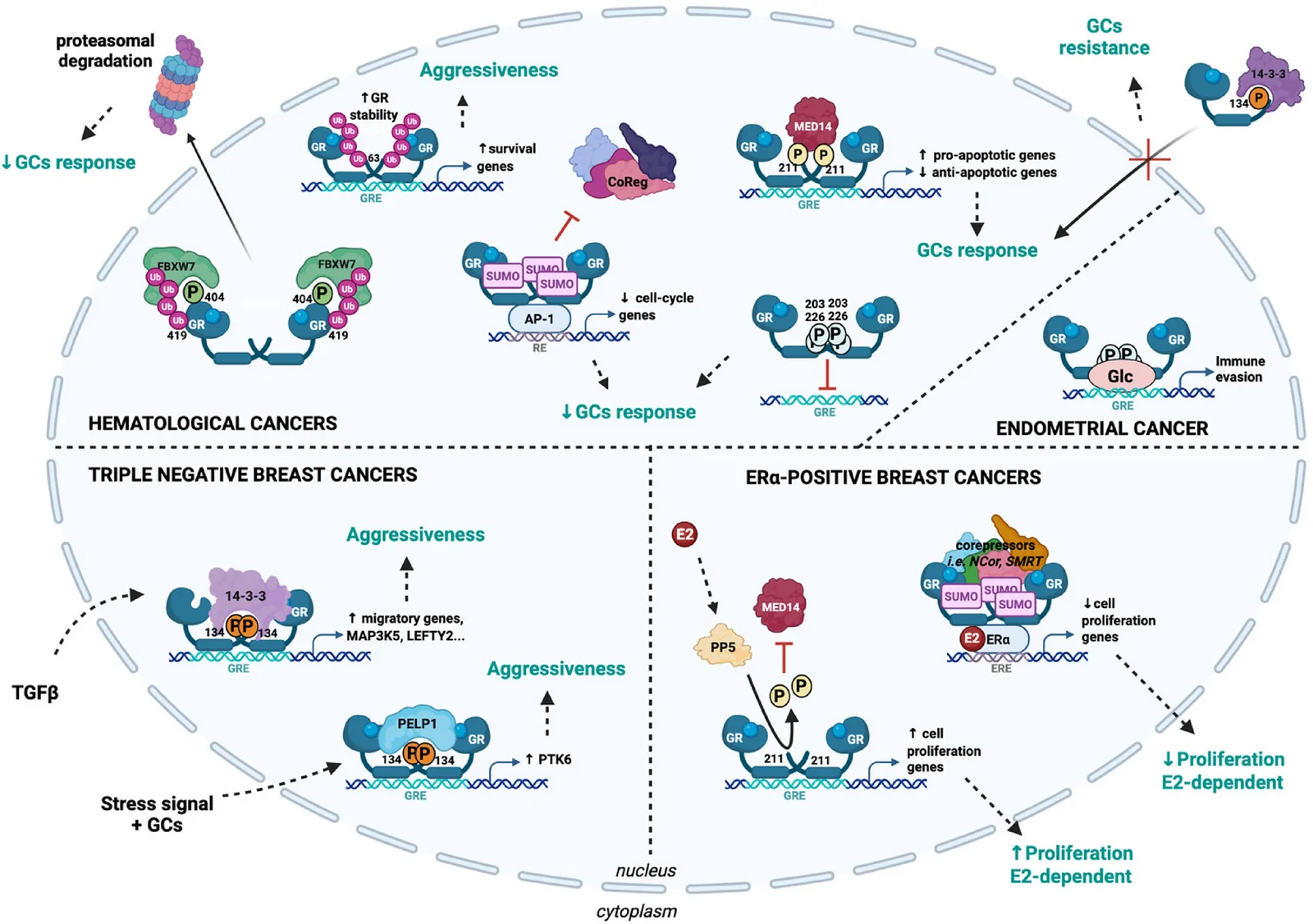
Involvement of GR PTMs in cancers. The figure is divided into different sections following the type of cancers and mechanisms associated. In hematological cancers, GR ubiquitylation, sumoylation and phosphorylation drive aggressiveness or decrease in GC sensitivity. In endometrial cancer, GR O-GlcNAcylation induces tumor evasion through the increase expression of PD-L1 and decrease expression of MHC-1. In triple negative breast cances, GR phosphorylation induces aggressiveness after recruitment of specific binding protein. In luminal breast cances, GR phosphorylation and sumoylation affects differently proliferation E2-dependent. GRE: GR response elements. P: phosphorylation, Glc: O-GlcNAcylation, Ub: Ubiquitylation

GCs are used as therapeutic agents in hematological cancers and as adjuvants in solid cancers to manage the side effects induced by chemotherapies. Owing to their broad action in cancers, many groups have studied the role of GR PTMs in cancer biology, deciphering a set of very specific mechanisms in each disease context (Fig. 3).

#### Hematological cancers

GCs are used as therapeutic agents in combination with other drugs in hematological cancers comprising acute lymphocytic leukemia (ALL), chronic lymphocytic leukemia (CLL), Hodgkin’s and non-Hodgkin’s lymphoma, in multiple myeloma. GCs trigger cancer cell death in hematological cancers, through the capacity of GR to upregulate pro-apoptotic genes (B-cell lymphoma 2 (BCL2), myeloid cell leukemia 1 (MCL1) and BCL2-like protein 1 (BCL2-L1)) and downregulate anti-apoptotic genes (BCL2-like protein 11 (BIM)) [169]. Additionally, in multiple myeloma, GCs inhibit c-Myc, an oncogene, and play a role in redox balance by promoting cell death. In childhood leukemia, GCs upregulate the thioredoxin interacting protein (TXNIP) which triggers reactive oxygen species (ROS) accumulation and leads to apoptosis [169].

Several GR PTMs have been described to play a role in leukemia. For example, pS211-GR is necessary for the induction of apoptosis in leukemia [130]. Likewise, in a GR-negative leukemia cell line (ICR-27), transfection of the GR S211A mutation was shown to reduce cell death compared to the WT GR [130]. In addition, in T-acute lymphoblastic leukemia (ALL), the inactivating mutation of FBXW7, the E3 ubiquitin ligase recruited by pS404-GR, regulating GR ubiquitylation and GR degradation, improves patient outcome and offers a better response to apoptosis induced by GC treatment [115].

In addition, studies have shown that targeting some phosphorylation events could modify response to GCs. For example, in T-ALL cell lines resistant to GCs, the inhibition of AKT1, which phosphorylates GR on S134, triggers a higher level of GR translocation to the nucleus and reverses a GC-resistant phenotype [90]. Indeed, AKT1 phosphorylation on S134 increases the binding of 14–3-3σ proteins to pS134-GR which reduces GR transcriptional activity by preventing the nuclear translocation of GR [91]. In B-cell acute lymphoblastic leukemia (B-ALL) cells, targeting ERK2 decreases GR phosphorylation on S203 and S226 [152]. This reduction of pS226-GR increases the recruitment of GR on chromatin and enhances target gene expression. Idelalisib, an ERK2 inhibitor that decreases pS203-GR and pS226-GR, enhances GC response in 90% of primary B-ALL patient samples [152]. In chronic lymphocytic leukemia (CLL), Ibrutinib, a Bruton’s tyrosine kinase (BTK) inhibitor and other PLCγ inhibitors, increases pS211-GR and decreases pS226-GR without affecting total GR levels, consistent with the activation of GR [158]. Ibrutinib increases the sensitivity to GCs in circulating CLL cells. However, in more integrated models, in microenvironmental conditions, JAK inhibitors, such as ruxolitinib, are necessary to inhibit ibrutinib-insensitive pathways [158].

Targeting sumoylation could also constitute a promising strategy to increase sensitivity to GCs. In B-ALL, inhibition of sumoylation increases GR occupancy on chromatin and subsequent changes in gene expression, resulting in the cell-cycle arrest induced by GCs [141]. *Ex-vivo* analyses on several patient samples showed a decrease in cell viability with the combination of GCs and TAK-981, a sumoylation inhibitor derived from ML-792 to be pharmacologically optimized [141, 168].

In multiple myeloma (MM), RNF6 expression is significantly higher than in the bone marrow of healthy donors [87]. RNF6 promotes polyubiquitylation of GR on K63. Surprisingly, it stabilizes the receptor and enhances its transcriptional activity, in order to promote tumor-cell proliferation and survival. These findings suggest that targeting RNF6 may represent a promising therapeutic strategy in multiple myeloma.

#### Breast cancer

Among all solid cancer types, breast cancer (BC) is the one in which most research on GR PTMs has been conducted. BCs are divided into different subtypes following the expression of three proteins: estrogen receptor (ERα), PR and epidermal growth factor receptor 2 (HER2). Luminal A and B BCs express ERα and PR, HER2-enriched BCs express high levels of HER2, and triple negative breast cancers (TNBCs) do not express any of these proteins and are associated with the poorest outcome.

In ERα-positive BC, GR expression is associated with a good prognosis because GR activation by GCs leads to apoptosis by modulating the expression of apoptotic genes. Moreover, GR interacts with ERα and leads to a favorable prognosis by blocking ERα-dependent cell proliferation [170]. The Rosenfeld group demonstrated that sumoylation at the three GR sites (K277, K293 and K703) is required for the repression of ERα gene transcription, as it promotes the recruitment of NCoR and SMRT, thereby inhibiting ERα-dependent proliferative effects [166]. In addition, it was demonstrated that estrogen decreases GR phosphorylation at S211 induced by GCs [134]. This decrease is associated with the fact that estrogen increases the expression of the phosphatase PP5, leading to the decrease in pS211-GR. As a result, cotreatment with estrogen and dex reduces pS211-GR levels, leading to a decrease in GR activity and attenuation of its associated anti-proliferative effects [134].

In TNBC, high GR expression was associated with poor outcome [170]. Indeed, GC induces an increase in cell migration, invasiveness, chemotherapy resistance and metastatic properties in TNBC [82, 171, 172]. Likewise, mifepristone treatment, a GR antagonist, increases caspase 3 and Poly (ADP-ribose) polymerase (PARP) cleavage and subsequently improves the efficacy of paclitaxel treatment [173].

Numerous reports from the Lange group showed that pS134-GR, highly expressed in TNBC is a key mediator of TNBC progression [93, 148–150]. This event promoted by p38 MAPK is induced in response to cellular stress [89]. Upon binding of dex, pS134-GR recruits PELP1 in order to activate the expression of the breast tumor kinase Brk/PTK6 [93, 150]. Using CRISPR knock-in models of TNBC cell lines expressing S134A-GR, Dwyer and colleagues show that pS134-GR is required for metastatic colonization of the lungs in female mice [149]. Comparing wild-type versus S134A GR TNBC cells, upon dex treatment, they identified specific subsets of target genes critical for TNBC migration and metabolism, such as PDK4 (pyruvate dehydrogenase kinase 4).

In addition, in TNBC cell lines, pS134-GR is necessary for the induction of cell migration mediated by transforming growth factor β1 (TGFβ1) [148]. Indeed, TGFβ1 treatment induces pS134-GR by p38 MAPK and the subsequent recruitment of 14–3-3ζ protein on this phosphorylation site which triggers the regulation of migratory genes. Interestingly, the authors identified a pS134-GR-dependent transcriptomic signature that comprises 24 genes induced by TGFβ1, predictive of poor survival in BC patients [148]. This signature includes key components of the p38 MAPK pathway, such as MAP3K5, required for intact p38 MAPK signaling.

Together, these data suggest a convergence between GR agonist, such as host stress or adjuvant GC prescribed, and cellular stress that induces pS134-GR, promoting aggressiveness of TNBC. Targeting pS134-GR in patients through its kinase (*i.e.,* p38 MAPK) or its binding partners (*i.e.,* PELP1 and/or 14–3-3ζ) could be of interest in TNBC. Indeed, a growing body of evidence implicates p38 MAPK and 14–3-3ζ in BC progression [174–176].

Mutation in *BRCA1* significantly increases the risk of BC, and 90% of these BCs belong to the TNBC subtype. In breast tissues from normal and TNBC cancer patients, either mutated or not for BRCA1, immunochemical analyses showed that pS211-GR was lost in BRCA1 mutated specimens compared to non-mutated specimens [177]. In cells, depletion of BRCA1 expression induces a decrease in dex-induced gene expression and the opposite was observed after BRCA1 overexpression. In addition, BRCA1 overexpression increased pS211-GR induced by p38 MAPK, as well as basal GR expression. Given that BRCA1 and GR do not interact, this suggests that BRCA1 modulates p38 MAPK, resulting in the modulation of GR activity through the increase in pS211-GR [177].

#### Other solid cancers

In uterine corpus endometrial cancer (UCEC), tumor cells reprogram their metabolism, leading to an increase in OGT activity. OGT interacts specifically with phosphorylated GR, and promotes its O-GlcNACylation at S132 [88]. This modification facilitates GR nuclear translocation and transcriptional activity. As a consequence, GR promotes the expression of programmed death ligand-1 (PD-L1) while repressing major histocompatibility complex class I (MHC-1). This effect impairs antigen presentation and allows tumor cells to evade immune detection. Disrupting the interaction between OGT and GR using competitive peptides decreases GR O-GlcNAcylation, decreases PD-L1 levels, and restores MHC-1 expression. The authors demonstrated that this was associated with improved anti-tumor responses mediated by CD8 + T cells both in vitro and in vivo [88]. These findings provide a potential explanation for the limited efficacy of immune checkpoint inhibitors in this subtype of endometrial cancer and supports O-GlcNAcylation signaling as a potential therapeutic target.

In non-small cell lung cancer (NSCLC), under basal conditions, GR predominantly associates with JNK in the cytoplasm. However, upon dex treatment, JNK is phosphorylated and activated, driving its interaction with GR in the nucleus. Surprisingly, this interaction stabilizes GR by preventing its ubiquitylation and subsequent proteasomal degradation. As a result, GR protein levels increase in NSCLC cells, influencing its transcriptional activity [178]. Patient data analyses further indicate that elevated expression of both GR and JNK is associated with a better prognosis in NSCLC.

Finally, recent work uncovered a link between GR ubiquitylation and the dissemination of colorectal cancer (CRC) cells [179]. Functional studies showed that knockdown of GR enhances colorectal cancer (CRC) cell proliferation, migration and invasion both *in vitro* and *in vivo*. Mechanistically, the authors showed that GR represses the expression of SETBP1, a gene associated with cancer progression. In CRC cells, mouse double minute 4 (MDM4) contributes to GR downregulation by facilitating its ubiquitylation and subsequent proteasomal degradation. Although MDM4 itself lacks intrinsic E3 ubiquitin ligase activity, it acts in cooperation with MDM2 to drive GR proteasomal degradation. Reduced GR levels then relieve SETBP1 repression, ultimately promoting CRC cell dissemination and progression [179].

### Neurological diseases

GR is broadly expressed across the brain, with particularly high levels observed in the hippocampus, hypothalamus and cortex [180]. Changes in GR activity, but also prolonged exposure to GCs, have been linked to various neurological and psychiatric disorders [181]. Studies using mouse models have also shown that GR plays an important role in emotional regulation and response to stress. Taken together, these data indicate that GR is a key regulator of brain function, and that its dysregulation may increase vulnerability to neurological disorders. In addition, several PTMs of GR, particularly phosphorylation events described below, have been associated with various disorders.

#### Depression and major depressive disorder (MDD)

Patients with MDD exhibit an imbalance in GR phosphorylation. In a study involving 30 MDD patients and 35 healthy controls, nuclear levels of total GR, pS211-GR and pS226-GR were measured in peripheral blood mononuclear cells. Specifically, pS226-GR, which suppresses transcriptional activity, increases, while pS211-GR, which promotes transcription, is comparatively less elevated. This leads to a decrease in the pS211-GR/pS226-GR ratio, indicative of a decrease in GR transcriptional activity, contributing to glucocorticoid resistance [165]. Specifically in women, higher pS226-GR levels were positively associated with neuroticism and self-reported symptoms of depression, anxiety and stress, whereas a lower nuclear pS211-GR/pS226-GR ratio was negatively associated with depressive symptoms [165]. In addition, chronic stress induced by social isolation in rat models, showed an increase in pS226-GR. Antidepressant treatment reduced this phosphorylation, supporting a role for pS226-GR in depression related to stress [182].

#### Alzheimer’s disease (AD)

AD is a global public health challenge that affects millions of elderly individuals, leading to cognitive decline and a reduced quality of life. AD is the most common form of dementia, marked by progressive memory loss, impaired cognitive function and the accumulation of amyloid-β plaques and a hyperphosphorylation of the Tau proteins in the brain. These pathological changes drive neurodegeneration. Aside from protein aggregation, abnormal cortisol levels and altered GR activation contribute to the age-related progression of AD [183].

BDNF (brain-derived neurotrophic factor) is critical for neuronal survival, synaptic plasticity and cognitive function. Using rat models, Lambert et al. showed that GCs paired or not with BDNF induce GR phosphorylation at S134 (rat S155) and S267 (rat S287). Mutations at these phosphorylated sites impair BDNF-mediated GR activation, leading to reduced expression of specific GR-target genes [94]. *In vivo*, authors demonstrated that GR phosphorylation is influenced by BDNF and TrkB levels, as well as stress [94]. Phosphorylation of GR at BDNF-responsive sites increased GR occupancy and cofactor recruitment at the promoter of BDNF-enhanced genes and is associated with dendritic spine formation and is modulated by chronic stress and antidepressant treatment [94, 184]. Disruption of GR phosphorylation at the BDNF-dependent sites in an APP/PS1 mouse model of early-stage Alzheimer’s disease worsened the detrimental effects of the APP/PS1 genotype on survival, neuroplasticity and cognitive function, while leaving amyloid-β deposition and vascular condition unchanged [183]. Loss of GR phosphorylation at BDNF-responsive sites via the MAPK phosphatase DUSP1, induced Tau hyperphosphorylation and dendritic spine loss in a mouse model. In the human cortex, DUSP1 expression is correlated with Tau phosphorylation, synaptic abnormalities, and cognitive decline in individuals with AD. It suggests that DUSP1 could be a promising therapeutic target for AD [151].

#### Bipolar disorder (BD)

BD is associated with abnormal GR signaling. Patients with BD display an overall decrease in GR phosphorylation but an increase in pS211-GR, particularly during depressive episodes. This dysregulation suggests a role for pS211-GR, a marker of activated GR, in BD pathophysiology [157].

#### Posttraumatic stress disorder (PTSD)

PTSD is a mental condition that can arise after exposure to extreme psychological trauma. It is characterized by chronic anxiety, recurrent nightmares and intrusive flashbacks. Li et al. demonstrated that the GR-FKBP51 complex increases in patients with PTSD, as well as in a mouse model of fear conditioning [156]. This alteration is accompanied by reduced pS211-GR, impaired nuclear translocation and lower expression of the GR-regulated gene 14–3-3ε. Treatment with a peptide that disrupts the GR-FKBP51 complex can restore GR phosphorylation on S211, nuclear translocation, enhance 14–3-3ε expression, and alleviate fear-related behaviors in mice [156].

#### Epilepsy

Epilepsy is a chronic neurological disorder characterized by frequent, unprovoked seizures. GCs have therapeutic benefits in epilepsy by activating anti-inflammatory responses, through the GR-annexin A1 (ANXA1) pathway. ANXA1 is produced following GR activation and nuclear translocation, to mediate anti-inflammatory effects [185]. Studies using temporal lobe epilepsy models show that during epileptogenesis and seizure progression, phosphorylated GR (pS211-GR) decreases in the hippocampus, and ANXA1 remains at baseline levels while inflammation appears, suggesting a lack of endogenous anti-inflammatory response [155]. Epileptic brain specimens exhibit abnormal ANXA1 expression [155]. Exogenous administration of ANXA1 partially reduces seizure activity, suggesting that impaired GR-ANXA1 signaling contributes to epilepsy [155].

#### Chronic social defeat stress (CSDS)

Lin et al. reported that CSDS is associated with a decrease in the expression of the SUMO E3 ligase PIAS1, while the levels of the other PIAS family members (PIAS2-4) remain unchanged [186]. They also showed that mice more susceptible to stress display reduced GR expression in the hippocampus, along with a global decrease in GR sumoylation, which coincides with a weaker interaction between PIAS1 and GR. This reduction in GR sumoylation was associated with increased binding of GR to GREs, suggesting an alteration of its transcriptional activity. Interestingly, overexpression of PIAS1 in the hippocampus was sufficient to restore stress resilience, whereas PIAS1 knockdown made mice more vulnerable to stress, even after a mild social defeat protocol [186]. Altogether, these results suggest that PIAS1-dependent sumoylation plays an important role in regulating GR function in the hippocampus, even without precise identification of the modification sites, and may influence responses to chronic stress.

### Inflammatory diseases

GR plays a central role in regulating immune responses and maintaining immune homeostasis. It is expressed in all major immune cell types, such as dendritic cells, macrophages, neutrophils and lymphocytes, where its controls inflammation, immune cell differentiation, migration, activation and survival. Importantly, GR activity is highly context- and dose-dependent and orchestrates both innate and adaptative immune responses [187, 188]. Although GR signaling is essential for maintaining immune homeostasis, its prolonged or excessive activation can lead to immunosuppression and increased susceptibility to infection [187]. Together, these findings place GR as a key factor in immune regulation and suggest that alterations in GR function, including its PTMs, may influence inflammatory disease severity and sensitivity to GCs. These mechanisms are discussed below.

#### Chronic obstructive pulmonary disease (COPD)

COPD is a chronic and progressive lung disease that obstructs airflow causing significant breathing difficulties often caused by long-term exposure to cigarette smoke. This chronic inflammatory disease is largely irreversible and GC-insensitive. Studies showed that defects in GR deacetylation contribute to GC insensitivity. Mechanistically, deacetylation of GR on K494 and K495 enhances the association between GR and the NF-κB complex, leading to the attenuation of pro-inflammatory gene expression in monocytes and lung cells [120]. Clinically, in lung tissue and alveolar macrophages of patients with COPD, a significant reduction in HDAC2 expression and activity have been observed, correlated with an increase in inflammation and the progression of the disease [167].

Studies demonstrated that p38 MAPK, which catalyzes GR phosphorylation on different sites, is overactivated in COPD lung macrophages [189]. P38 MAPK inhibitors were shown to reduce inflammatory-related mediator release from alveolar macrophages of patients with COPD [190]. Likewise, in peripheral blood mononuclear cells (PBMCs) from patients with COPD, inhibition of p38 MAPK restores GC sensitivity by reducing dex-induced activation of pS211-GR in PBMCs [153].

#### Severe asthma (SA)

Severe asthma (SA) is characterized by GC insensitivity due to a defect in GR function. Different reports demonstrated a dysregulation of phosphatases in SA. In ASM cells from severe asthmatic patients, fluticasone, an inhaled GC, failed to induce GR phosphorylation on S211 and specific GR target genes (*i.e.,* GILZ). In airway smooth muscle (ASM) cells from healthy and severe asthmatic patients, this insensitivity was explained by a high expression of the phosphatase PP5 in severe asthmatic patients. Likewise, PP5 expression was higher in the ASM bundles in endobronchial biopsies of severe asthmatic patients [154].

In PBMCs from patients with SA, Kobayashi et al. showed that the expression and activity of PP2A were reduced compared with healthy volunteers, resulting in an increase in the phosphorylation of GR at S226 and activation of JNK1, one of the kinases responsible for this modification [159]. Other studies demonstrated a key role of pS226-GR in SA, linked with p38 MAPK, another enzyme catalyzing pS226-GR. In bronchial epithelium of SA patients, p38 MAPK levels and pS226-GR are higher, linked with GC insensitivity [107, 160–162]. Interleukin IL-2 and IL-4, which are overexpressed in the airways of patients with SA, were shown to reduce GR nuclear translocation in T cells by inducing phosphorylation of pS226-GR through the p38 MAPK pathway [162–164]. This effect can be reversed by the p38 MAPK inhibitor without affecting pS211-GR [107, 161].

### Reproductive disorders

GR is ubiquitously expressed across the female reproductive system where it contributes to the regulation of fertility, reproduction, placental function and fetal development [191]. In the ovary, GR signaling participates in several processes by modulating the activity of granulosa cells, oocytes, cumulus cells and luteal cells [192]. Within the uterus, GR regulates cell proliferation by regulating some uterine gene expression and antagonizes estrogen-dependent growth [193, 194, 195]. GR activity is also required during pregnancy, particularly for the initiation of the labor [196]. GR contributes to development of the mammary gland during puberty and pregnancy, and prevents excessive apoptosis during lactation [197, 198, 199]. In addition, GCs suppress the reproductive function by reducing gonadotropin-releasing hormone (GnRH) pulsatility and pituitary responsiveness, thereby suppressing gonadotropin secretion, particularly luteinizing hormone (LH) [142, 200]. Using a murine gonadotrope cell line (LβT2) and mouse pituitary explants, it was demonstrated that GCs rapidly interfere with GnRH signaling through a non-genomic pathway mediated by GR palmitoylation [124]. Activation of this signaling triggers rapid phosphorylation of Ca2 +/calmodulin-dependent protein kinase II (CaMKII) and synapsin-1 within minutes, an effect sensitive to inhibition of protein palmitoylation. Functionally, GC signaling attenuates GnRH-induced CaMKII activation, leading to reduced LH release from pituitary explants [124].

### Metabolic disorders

GR is a central regulator of metabolic homeostasis, by regulating genes involved in glucose production, storage and use. For example, GR stimulates gluconeogenesis, through increased expression of key enzymes such as phosphoenolpyruvate carboxykinase (PEPCK) and glucose-6-phosphate (G6Pase) [201]. In skeletal muscle and white adipose tissue, GR also promotes the expression of glucose transporters, such as GLUT4, facilitating glucose uptake and storage. In adipose tissue, GCs stimulate the expression of lipogenic genes, such as sterol regulatory element-binding protein 1 (SREBP-1), which promotes the synthesis of fatty acids and triglycerides. Through these processes, GCs help to maintain metabolism and energy homeostasis in response to stress, such as starvation and exercise. The importance of GR signaling in metabolism is highlighted by endocrine disorders. Excess GC exposure, as observed in Cushing’s syndrome, is linked to obesity, hyperglycemia and hepatic steatosis, whereas GC deficiency in Addison’s disease results in weight loss and hypoglycemia [202]. Under chronic stress and prolonged GC treatment, the persistent activation of GR has been associated with metabolic disorders, although the contribution of GR PTMs to these effects remains poorly characterized.

A study demonstrated that AMP-activated protein kinase (AMPK), a key regulator of cellular energy homeostasis, modulates GC signaling via the activation of p38 MAPK [132]. Activated p38 MAPK enhances GR phosphorylation at S211, which regulates GR-dependent transcription in a tissue- and promoter-specific manner by altering the recruitment of transcriptional coregulators. In vitro and in vivo analyses showed that AMPK activation reverses several metabolic consequences of dex treatment, including hepatic steatosis, elevated blood glucose and increased hepatic glycogen accumulation. These effects could be attributed to the inhibition of the gluconeogenic enzymes, PEPCK and G6Pase [132].

More recently, the ubiquitin E3 ligase seven-in-absentia homolog 2 (SIAH2) was identified as a new regulator of GR function in white adipose tissue during GC exposure [203]. Using Siah2 knockout mice, the authors showed that loss of SIAH2 increases GC-induced adipocyte hypertrophy and is associated with increased expression of genes linked to fibrosis and inflammatory pathways. Mechanistically, SIAH2 regulates GR signaling at two distinct levels: it controls GR protein abondance through its enzymatic ubiquitin ligase activity, while modulating GC-dependent GR transcriptional activity through a mechanism that is independent of its enzymatic function [203].

### Erythroid disorders

GR signaling plays an important role in erythropoiesis by promoting the proliferation and survival of both early erythroid progenitors and more differentiated precursors involved in red blood cell production. Clinically, synthetic GCs such as dex are used to stimulate erythropoiesis in disorders like Diamond-Blackfan anemia (DBA) and pediatric aplastic anemia, while prednisone has also been shown to enhance erythroid production even in non-anemic individuals [204].

A recent work demonstrated that NLK-mediated phosphorylation of GR in S226 limits erythropoiesis by promoting its ubiquitin-dependent proteasomal degradation [108]. NLK is highly expressed in committed erythroid progenitors and hyperactivated in erythroid progenitors of DBA patients [205]. Inhibition of kinase activity of NLK using the specific inhibitor OTS167 decreases GR phosphorylation on S226, resulting in enhanced proliferation of early erythroid progenitors, which in turn increases erythroid differentiation of erythroprogenitor cells [108]. Targeting NLK activity could offer a new therapeutic strategy.

## Conclusion and future directions

This review highlights the growing complexity of GR regulation by PTMs and their involvement in human pathologies, keeping in mind that the field is still in its infancy. An increasing number of studies demonstrate that aberrant GR PTMs are associated with diseases, and emerging evidence indicates that pharmacologically targeting the enzymes that write, erase, or read these PTMs may have therapeutic benefits (Table 2). By integrating mechanistic insights and clinical observations, this review underscores the relevance of GR PTMs as novel leverage points to improve GC efficacy, while limiting adverse effects. This topic is particularly timely due to the expanding clinical application of GCs and GR antagonists across nearly all fields of medicine.

A striking feature of GR regulation is that the majority of PTM sites, particularly phosphorylation sites, are located within the intrinsically disordered N-terminal domain containing AF-1 (Fig. 1). It was suggested that the intrinsically disordered protein (IDP) of this region enables GR to assess its molecular environment until appropriate binding partners are encountered [206]. Such structural plasticity likely allows PTMs to dynamically reconfigure interaction surfaces, thereby influencing GR conformation, coregulator recruitment and transcriptional output. This is highlighted by the number of readers identified for GR PTMs in this region (Fig. 1C). Understanding how GR PTMs operate within this intrinsically disordered framework represents a central challenge for the field.

While GR phosphorylation remains the most extensively studied PTM, this review highlights a growing list of additional modifications, such as sumoylation, ubiquitylation, acetylation, palmitoylation and O-GlcNAcylation, the implication of which is increasing in pathological processes. However, much of the current knowledge is derived from *in vitro* studies, often using cancer cell models. In contrast, *in vivo* evidence, particularly from patient samples, is mainly confined to neurological and inflammatory disorders. Expanding the analysis of GR PTMs to more disease contexts and clinically-relevant tissues will be essential to establish their causal contributions to pathophysiology. However, technical limitations remain a major barrier. The lack of site-specific antibodies and engineered models, such as knock-in mice for specific GR PTMs, currently limits precise dissection of individual PTM functions *in vivo*.

Importantly, an additional and often underappreciated layer of complexity arises from PTMs of GR coregulators themselves. These PTMs regulate their interactions with GR, its subcellular localization, conformation, and enzymatic activity, thereby modulating GR transcriptional responses [35, 207]. For example, the coactivator activity of the lysine methyltransferase G9a/GLP is tightly controlled by PTMs. G9a/GLP self-methylation promotes recruitment of HP1γ and enhances coactivator function, whereas phosphorylation by Aurora kinase B disrupts this interaction and represses activity. These regulatory mechanisms influence specific GC-regulated processes, including lung cancer cell migration, GC-induced apoptosis in B-cell lymphoblastic leukemia, or metabolic responses such as insulin resistance [83, 208, 209]. Given that the majority of PTMs affecting coregulators remain to be identified and characterized, their integration into GR signaling networks represents a major obstacle in the field.

Important tissue-specific knowledge gaps in pathology also persist. GR plays an essential role in the cardiovascular system, regulating cardiac development, blood pressure, heart function and vascular health. Dysregulation of GR signaling has been associated with diseases such as atherosclerosis, heart failure, sepsis and myocardial ischemia [210]. However, despite the recognized importance of GR in cardiovascular physiology, the contribution of GR PTMs to these pathologies remains largely unexplored. Some PTMs, such as GR sumoylation or ubiquitylation associated with receptor degradation, seem to have broad pleiotropic effects. This suggests that some GR PTM-dependent mechanisms may be shared across multiple organ systems.

Another major gap concerns sex as a biological variable. With few exceptions, such as breast or endometrial cancer, GR PTM research has predominantly relied on male-based models. Emerging evidence indicates that response to GR PTMs can differ following the sex. For example, female placentae display higher basal levels of OGT and OGT/GR complex compared to males [211]. Sex-dependent differences in the machinery regulating GR O-GlcNAcylation appear to contribute to distinct male and female responses to stress, highlighting the importance of studying GR PTMs in a sex-specific context.

In the long term, advances in large-scale technologies should advance the field. Mass spectrometry-based proteomics will enable the comprehensive identification and quantification of GR PTMs across different tissues and physiological conditions, while interactome approaches will provide a better understanding of the protein networks associated with these PTMs. Combined with emerging genome editing tools and improved *in vivo* models, these approaches will facilitate our understanding of how PTMs orchestrate GR function at the global level.

Taken together, the emerging picture is that GR PTMs constitute a multilayered and context-dependent regulatory code that integrates multiple signals. Deciphering this code, through improved molecular tools, *in vivo* models, patient-based studies and sex-inclusive research, will be essential for translating GR PTM biology into innovative and more precise therapeutic strategies.

## Data Availability

No datasets were generated or analysed during the current study.
